# Pollutant dispersion by tall buildings: laboratory experiments and Large-Eddy Simulation

**DOI:** 10.1007/s00348-022-03439-0

**Published:** 2022-05-23

**Authors:** H. D. Lim, Denise Hertwig, Tom Grylls, Hannah Gough, Maarten van Reeuwijk, Sue Grimmond, Christina Vanderwel

**Affiliations:** 1 Aeronautics and Astronautics Department, University of Southampton, Southampton, UK; 2 Department of Meteorology, University of Reading, Reading, UK; 3 Department of Civil and Environmental Engineering, Imperial College London, London, UK

## Abstract

**Abstract:**

Pollutant dispersion by a tall-building cluster within a low-rise neighbourhood of Beijing is investigated using both full-scale Large-Eddy Simulation and water flume experiments at 1:2400 model-to-full scale with Particle Image Velocimetry and Planar Laser-Induced Fluorescence. The Large-Eddy Simulation and flume results of this realistic test case agree remarkably well despite differences in the inflow conditions and scale. Tall buildings have strong influence on the local flow and the development of the rooftop shear layer which dominates vertical momentum and scalar fluxes. Additional measurements using tall-buildings-only models at both 1:2400 and 1:4800 scales indicates the rooftop shear layer is insensitive to the scale. The relatively thicker incoming boundary layer affects the Reynolds stresses, the relative size of the pollutant source affects the concentration statistics and the relative laser-sheet thickness affects the spatially averaged results of the measured flow field. Low-rise buildings around the tall building cluster cause minor but non-negligible offsets in the peak magnitude and vertical location, and have a similar influence on the velocity and concentration statistics as the scale choice. These observations are generally applicable to pollutant dispersion of realistic tall building clusters in cities. The consistency between simulations and water tunnel experiments indicates the suitability of both methodologies.

**Graphical abstract:**

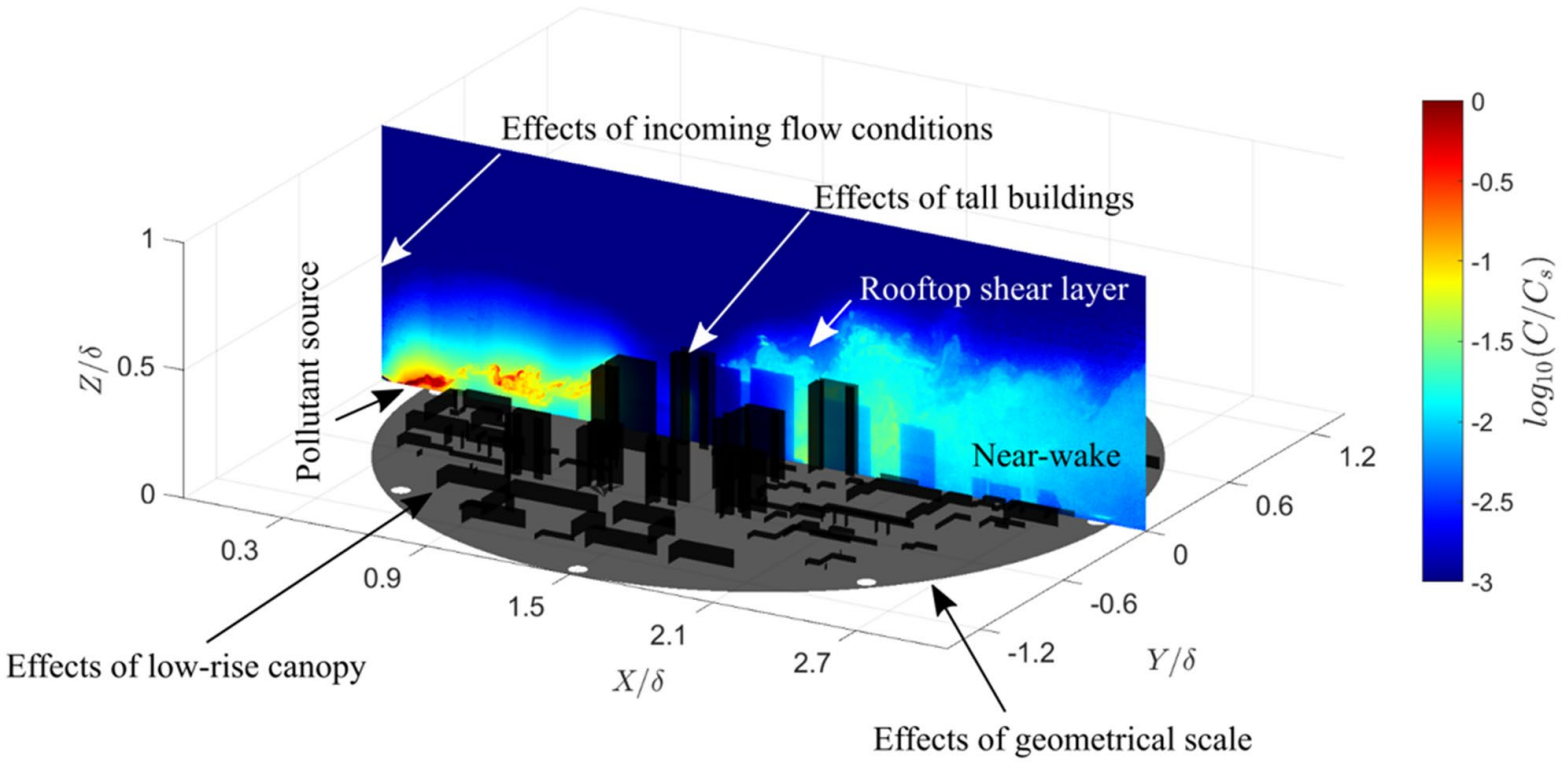

## Introduction

Urban landscapes worldwide are extending vertically. The demand for high-rise commercial centres and tall residential buildings is increasing in regions such as Asia due to the scarcity of land and the need to house an increasing urban population close to their workplaces. Buildings have a profound effect on near-surface atmospheric processes like airflow and scalar transport (Britter and Hanna 2003; Belcher 2005). Tall buildings, whether isolated or in clusters, alter and exacerbate such effects.

**Fig. 1:**
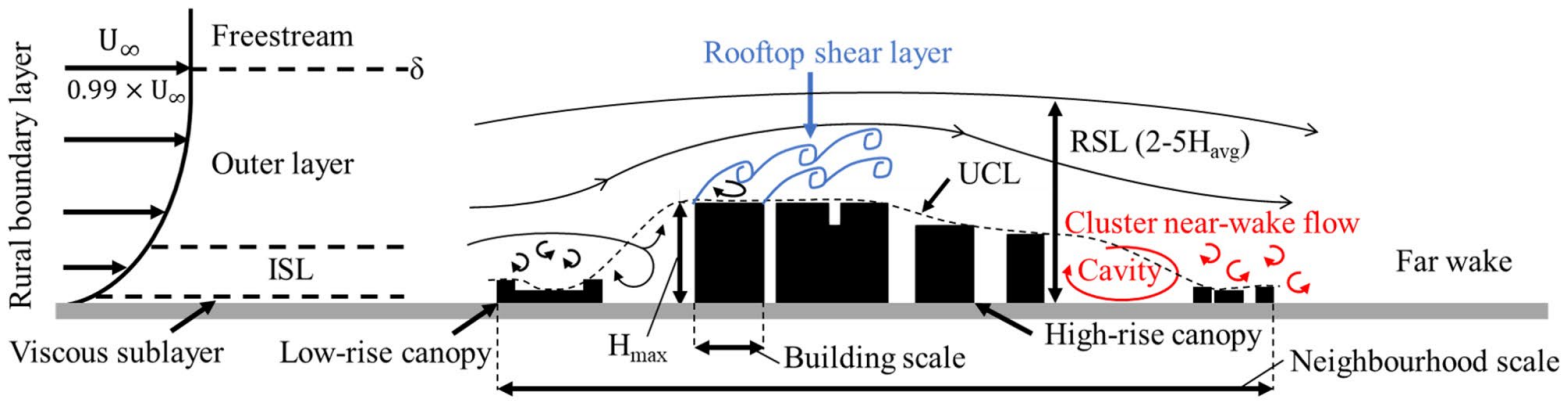
Dominant flow features of an atmospheric boundary layer flow through an urban neighbourhood. Sketch is not to scale. ISL: Inertial sublayer, RSL: roughness sublayer, $H_{avg}$: average building height, $H_{max}$: maximum building height, $U_{\infty }$: freestream velocity, $\delta$: boundary layer thickness

Tall buildings exert their influence on urban processes over a range of spatial scales, from impacting surroundings buildings (e.g. pressure distributions on façades) and street canyons (flow-regime changes) to neighbourhoods in the building wake (drag distribution). The subregions of the atmospheric boundary layer and the dominant flow features within an urban neighbourhood relevant to both building and neighbourhood scales are sketched in Fig. 1. At the building scale, the momentum and scalar fluxes in the roughness sublayer (RSL) are intricately connected to the urban morphology. Some insights into this can be gained by considering studies of cuboids (i.e., finite-length square cylinders). For an isolated cube, Oke et al. (2017) describe how the initial flow field diverges in the vertical and lateral directions to form recirculating flows on the roof and side walls which reattaches if the building length in the direction of the flow is greater than its height. The lee of the building is dominated by flow separation, with the formation of a cavity zone (recirculating flow) and turbulent wake. Near the ground, horseshoe vortices wrap around the building resulting in high turbulence intensities that define the lateral boundaries of the wake (Simpson 2001; Oke et al. 2017).

The height-to-diameter aspect ratio (AR) of isolated obstacles has a strong influence on the resulting flow field. For an isolated finite-length round cylinder, Tanaka and Murata (1999) describes how the edge vortices (shed from the sides of the cylinder) interact with the free-end vortex (shed from the tip), forming the ‘legs’ and ‘heads’ of the ‘arch’-type vortices in the wake, respectively. While the arch-type vortices match the outline of the cylinder at $AR=10$, lower aspect ratio cylinders ($AR\le 2.5$) have arch vortices with legs that stretch outwards in the transverse direction and could extend to several diameters from the cylinder. Base and tip vortices associated with the bending of the arch-type vortex system are also observed in the near-wake of cuboids (Wang et al. 2006; Wang and Zhou 2009). These vortices are closely related to upwash and downwash effects that can exchange air pollutants between the near-wake flow and freestream.

At the neighbourhood scale, studies of isolated tall buildings within a low-rise neighbourhood have document the strong impact on the vertical exchange of turbulent and advective momentum and scalars (e.g. Fuka et al. 2018). Flow and scalar fields in the low-rise canopy are affected by the tall building over large distances (Brixey et al. 2009; Heist et al. 2009). Likewise, roughness effects of the underlying low-rise canopy can alter the dynamics of the wake zone of tall buildings by breaking down larger vortices (Hertwig et al. 2019), thus destroying some of the organised motions that have been extensively documented for flow past wall-mounted bluff bodies (e.g. Castro and Robins 1977; Hunt et al. 1978). Figure 1 illustrates this scale interaction in the context of an oncoming rural turbulent boundary layer impacting a tall-building cluster within a low-rise neighbourhood. In this, the upstream low-rise canopy influences the incoming flow that impacts the tall building to create the rooftop shear layer, and the tall buildings’ wake interacts with the downstream low-rise canopy to alter the cluster near-wake dynamics.

Such scale interactions represent a challenge for urban land surface modelling. City-wide neighbourhood scale approaches to modelling the urban canopy layer (UCL) and urban air pollution do not resolve the buildings explicitly, but instead rely on concepts applicable to simple canonical flows like rough-wall boundary layers or two-dimensional street-canyons of uniform height (Masson 2006; Grimmond et al. 2009), in which dynamics in the UCL and RSL are decoupled from processes in the inertial sublayer (ISL), which is assumed to be well-developed. Next-generation regional numerical weather prediction models will have resolutions of ${\mathcal {O}}$(100 m) or higher and hence operate within the building grey-zone (Barlow et al. 2017; Lean et al. 2019), in which the grid resolution approaches the building length scale(s). Sub-grid scale variability becomes large and bulk urban morphology characteristics can differ vastly between grid-boxes (Kanda et al. 2013; Kent et al. 2019).

Large variations in building heights (e.g. tall buildings within a low-rise neighbourhood) extend the vertical structure of the UCL but also make its depth less well defined. Aerodynamic roughness length and displacement height used in classic similarity frameworks are typically derived from methods assuming idealised uniform-height building arrays (e.g. Macdonald et al. 1998). The Kanda et al. (2013) modification recognises the impact of tall buildings and building-height variability on the displacement height in realistic urban settings, resulting in displacement heights that are much larger than derived with conventional methods (Kent et al. 2017). Sützl et al. (2021) show that dispersive fluxes in the RSL become important in spatially-averaged budgets when strong vertical and horizontal heterogeneity in an urban area exists, supporting the argument that dispersive stresses should be represented to improve urban canopy models (e.g. Giometto et al. 2016).

Such model development needs can only be addressed by having suitable data to derive and test parametrisations. Turbulence-resolving Computational Fluid Dynamics (CFD) methods, like Large-Eddy Simulation (LES), can provide valuable insight into the spatio-temporal structure of urban flow fields (e.g. Letzel et al. 2008; Tolias et al. 2018), but model output needs to be evaluated carefully through comparison with suitable observations. Complementary to CFD approaches, laboratory experiments using scale models in low-speed wind tunnels or water flumes are valuable for urban flow and dispersion studies, as they offer flexibility in model design and full control of inflow and boundary conditions. The reduction in physical complexity (*cf.* field observations) make laboratories ideal for systematic studies of urban processes (e.g. Baik et al. 2000; Pournazeri et al. 2012; Di Bernardino et al. 2015). However, laboratory studies involve uncertainties inherent to the facility type, measurement technique or choice of model scale (e.g. Wang et al. 1996; Saathof et al. 1995; Kanda 2006), which have to be quantified and considered carefully when analysing and interpreting the results (Torres et al. 2021).

This study explores the important features of the flow and dispersion around a cluster of tall buildings surrounded by a low-rise canopy, using a Beijing neighbourhood as a case study (Sect. 2.1). Uniform arrays of buildings have been studied in the past, but the results are not always applicable to realistic urban geometries (Vanderwel and Ganapathisubramani 2019), hence motivating this study as cities become increasingly critical to simulate for a wide range integrated urban services (Grimmond et al. 2020). The few studies that have considered realistic urban settings with tall building clusters have mainly focused on wind loads (Nozu et al. 2015; Yan and Li 2016; Elshaer et al. 2017). Here we use both water-flume experiments (Sect. 2.2) and LES (Sect. 2.3) to demonstrate their capabilities in a realistic scenario and to explore the effect of the tall-building cluster on the flow structure and scalar transport (Sect. 3).

We use this case study to explore fundamental questions relating to the methodologies and physical processes of realistic urban geometry flow. Although thermal stratification and differential heating have been demonstrated to influence flow and dispersion in idealised urban environments studies (Nazarian et al. 2018; Marucci and Carpentieri 2020), in this more realistic geometry study we only consider neutral atmospheric stability (i.e., Richardson number is close to zero), and therefore negligible buoyancy effects. In Sect. 3.1, we focus on the scale dependency of the flume results. In Sect. 3.2, we explore the physical flow structure and scalar distribution in the cluster wake and discuss the suitability and limitations of flume experiments and LES. In Sect. 3.3, we investigate the impact of low-rise neighbourhood buildings on the cluster wake in the flume. In our conclusions section (Sect. 4), we discuss that our findings provide important and applicable results for real cities that are of concern for weather extremes and climate change; as well as make recommendations for future scaled experiments and LES methodologies.

## Methodology

### Study area

**Fig. 2:**
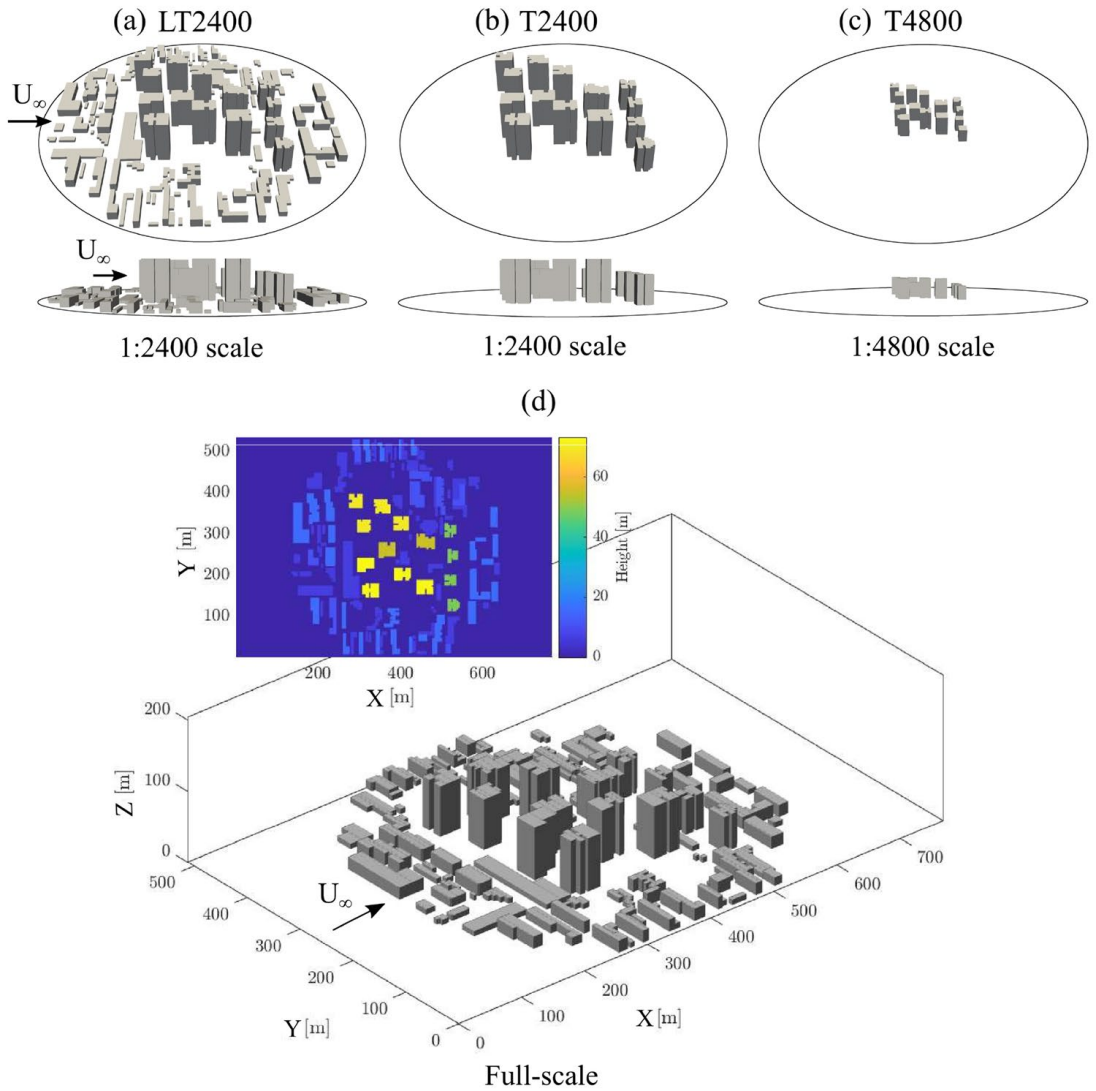
3D-printed reduced-scale urban models used in the water-flume (circle indicates model ground plate extent): **a** LT2400, **b** T2400 and **c** T4800; and **d** LES at full-scale with computational domain for LT2400. For each case the inflow ($U_{\infty }$) is from the North (arrows)

The Haidian District is a typical residential–commercial area in Beijing, with a cluster of 14 tall buildings surrounded by a low-rise neighbourhood. To characterise this area in the laboratory experiments, we used three scale models (Fig. 2a–c) with a test area of $\sim$580 m full-scale extent (for details see Hertwig et al. 2021). This neighbourhood was represented by 3D-printed models (Hertwig et al. 2020) which contain all main building structures with small geometrical simplifications at two geometric scales (1:2400 and 1:4800):

1. **LT2400** (1:2400 model scale; Fig. 2a) is a detailed model of low-rise buildings and the tall-building cluster. The low-rise buildings are binned at three heights based on a modal analysis of the neighbourhood: $H_{min,LT2400}$ = 2 mm (4.8 m at full-scale), 4 mm (9.6 m), and 6 mm (14.4 m). The height of the tall elements is between 20 mm (48 m) to $H_{max,LT2400}$ = 30 mm (72 m).
2. **T2400** (1:2400; Fig. 2b) only has the tall building cluster without the low-rise surroundings. The height of the tall elements is between $H_{min,T2400}$ = 20 mm (48 m) to $H_{max,T2400}$ = 30 mm (72 m).
3. **T4800** (1:4800; Fig. 2c) uses the same geometry as T2400, but at a reduced scale so that the heights of the tall elements range between $H_{min,T4800}$ = 10 mm (48 m) and $H_{max,T4800}$=15 mm (72 m).

**Fig. 3:**
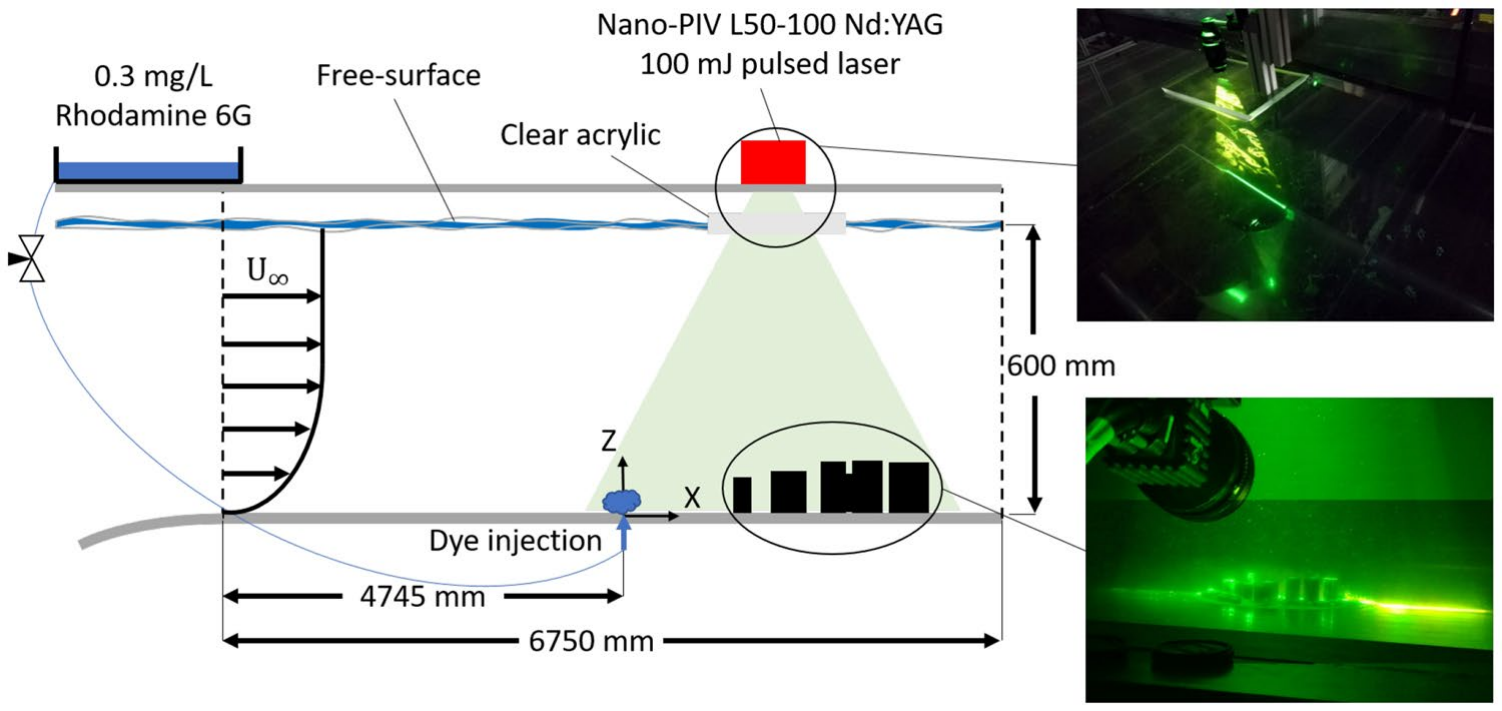
Particle Image Velocimetry (PIV) and Planar Laser Induced Fluorescence (PLIF) setup in the water flume. Images (right) show the laser sheet directed through an acrylic sheet mounted at the water surface onto the 3D-printed urban model

In the LES, the modelled buildings are the same as the LT2400 model (Fig. 2d) but simulations are performed at full-scale. For both the flume and the LES data, results are presented using a Cartesian coordinate system in which *X* is the streamwise, *Y* the lateral and *Z* the vertical direction (Figs. 2d, 3), with corresponding instantaneous velocities *u*, *v* and *w*. Time-averaged variables are given in uppercase such that, e.g., $u = U + u'$, where the prime indicates the fluctuation about the mean.

### Water tunnel experimental methodology

The University of Southampton’s water flume facility has a test section that is 6,750 mm long and 1,200 mm wide (Fig. 3). Throughout the measurement campaign, the water depth and free-stream velocity are maintained at a constant level of $600\pm 1$ mm and $U_{\infty \mathrm {EXP}}=0.46$ m s$^{-1}$. The 3D urban model is fixed to a smooth acrylic ‘false floor’ that overlies the entire length of the test section glass bottom. Building configurations analysed in this study are aligned to have an inflow direction from North.

**Fig. 4:**
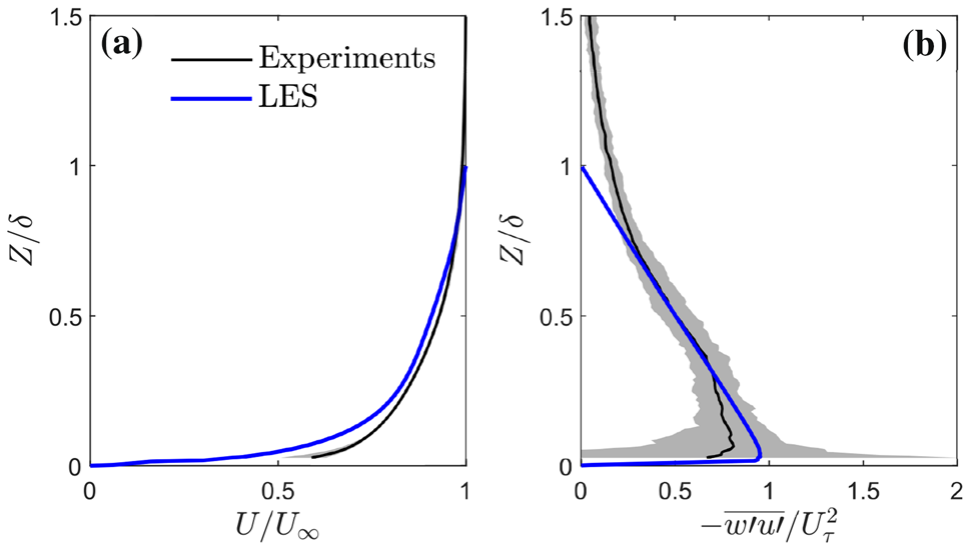
Water flume and LES incoming flow vertical profiles (time- and spatial-mean) of: **a** streamwise velocity and **b** Reynolds shear stress with 5th and 95th percentile (shading) bounds of experimental data. Flume and LES data are normalized by their respective $U_{\infty \mathrm {EXP}}$, $U_{\tau \mathrm {EXP}}$, $\delta _{\mathrm {EXP}}$, $U_{\infty \mathrm {LES}}$, $U_{\tau \mathrm {LES}}$ and $\delta _{\mathrm {LES}}$

Vertical profiles of the incoming flow (Fig. 4) fit a power law description of the boundary layer, $U/U_{\infty \mathrm {EXP}}=(Z/\delta _\mathrm {EXP})^\alpha$, with an exponent of $\alpha \approx$0.11, which is typical of rural or rural-to-urban terrain flows (Tomas et al. 2017). The incoming flow boundary layer thickness is $\delta _\mathrm {EXP}$=83 mm based on the definition of 0.99$U_\infty$. The friction velocity is $U_{\tau \mathrm {EXP}}$=0.0173 m s$^{-1} \pm 10\%$ (Appendix A). The logarithmic region is found to be valid from approximately $0.04< Z/\delta _\mathrm {EXP} < 0.22$, consistent with smooth wall turbulent boundary layers.

To reproduce the turbulence scales of full-scale sharp-edged buildings (fixed separation points) and achieve Reynolds number independence, the Reynolds number (based on building height) should be greater than 5,000–10,000 (Plate 1999). Here, based on the tallest building height and $U_{\infty \mathrm {EXP}}$, $Re\approx$ 14,000 for the 1:2400 scale model and $Re\approx$ 7,000 for the 1:4800 scale model. These should be sufficiently large to reproduce the turbulent characteristics of the full-scale flow.

Two-dimensional particle image velocimetry (PIV) and planar laser induced fluorescence (PLIF) measurements are performed simultaneously to obtain velocity and concentration measurements in the streamwise plane. The spanwise position (Y location) of the measurement plane is at the flume’s centre-plane (i.e., at maximum distance from the flume’s side walls) to avoid any potential flow asymmetry due to the boundary layers of the side walls. For the wind direction analysed here, the plane is located in the middle of one of the tallest building to investigate its influence on the wake flow (Fig. 2). For the PIV measurements (Appendix B), the flow is seeded with 50 $\mu$m polyamide seeding particles, and allowed to recirculate in the flume until the desired seeding density and uniform particle distribution is achieved. Illumination is provided by a 100 mJ Nd:YAG double-pulsed laser with an emission wavelength of 532 nm operated at 4 Hz. Two 4 MP CMOS cameras are used in a side-by-side configuration to achieve a field-of-view of 230 mm streamwise $\times$ 135 mm vertical. LaVision’s Davis 8.4.0 software (LaVision GmbH 2019) is used to acquire and process the images.

For the PLIF measurements, a neutrally buoyant solution of Rhodamine 6G fluorescent dye with concentration levels of $C_S$=0.3 mg L$^{-1}$ is continuously introduced to the flume at ground level, 5 mm upstream of the model plate using a 3 mm diameter tube. The dye flow rate is maintained at a constant rate (30 cm$^3$ min$^{-1}$) by a needle valve to ensure minimal disturbance to the flow in the flume. The dye Schmidt number (Sc=2500±300, (Vanderwel and Tavoularis 2014)) indicates momentum diffuses at a much higher rate than the scalar. The absorption and emission peaks of Rhodamine 6G are 525 nm and 554 nm, respectively. An optical long-pass filter with a sharp cutoff (540 nm) is used to block out incident light from the laser and reflected light from the PIV particles. This filter is in front of the 5.4 MP 16-bit depth sCMOS camera used to record the fluorescence emitted from the excited dye. Local concentrations are determined from the fluorescence intensity in a calibration procedure (Appendix C).

### LES methodology

The uDALES model (Tomas et al. 2015; Grylls et al. 2019, ][) is a high-resolution, large-eddy simulation (LES) code for simulation of the urban environment atmospheric boundary layer. LES models resolve the flow at the energetically dominant scales of turbulent motion by filtering the Navier-Stokes equations. They are therefore able to resolve the unsteady nature of turbulent flows unlike Reynolds-averaged Navier-Stokes models while being less computationally intensive than direct numerical simulation techniques. LES provides an optimal tool to model the spatial and temporal scales necessary to investigate both the urban micro-scale environment and the atmospheric boundary layer.

uDALES is adapted from the Dutch Atmospheric Large-Eddy Simulation model (DALES) (Heus et al. 2010). Buildings are modelled using the immersed boundary method (Tomas et al. 2015). Log-law wall functions are implemented to capture the near-wall dynamics (Uno et al. 1988; Suter 2018). The gradient diffusion hypothesis is used to close the subgrid scale terms following the approach of Vreman (2004) and Suter (2018). uDALES uses finite differences with variables spatially discretised on an Arakawa C-grid. A second-order differential scheme is applied to all field variables except the pollutant fields. The latter uses a kappa scheme to ensure positivity. A third-order Runge-Kutta time integration scheme is applied (Heus et al. 2010; Grylls 2020).

The flow conditions of the flume experiments just upstream of the building cluster are reproduced in an approximate manner by using a driver simulation with a domain size of 768$\times$512$\times$203 m (Table 1). The vertical height of the domain matches the estimated boundary layer depth of the 1:2400 scale water tunnel experiment ($\delta _\mathrm {EXP} \approx$ 83 mm) at full scale ($\delta _{LES} = 203$ m). Periodic boundary conditions are employed for the lateral boundaries. A free-slip condition is applied at the top of the domain and wall functions are applied at the façades of the immersed boundaries. Neutral atmospheric conditions are enforced. The simulation is forced by a constant pressure gradient. A spin-up period of 122,400 s is used to allow the flow field to reach a statistical equilibrium, after which data are analysed for 28,800 s. During this period, the velocity fields at the outlet plane ($X = 768$ m) are saved every second for use as the inlet boundary condition for the verification simulation (Table 1).

**Table 1:** LES parameters for the driver and verification simulations, where *P* indicates periodic boundary conditions (BC) and *I* inflow-outflow BC

Simulation	Grid size	Domain size	$U_{\tau \mathrm {LES}}$	Run-up	Averaging period	*X* BCs	*Y* BCs
	[-]	[m]	[m s$^{-1}$]	[s]	[s]		
Driver	576$\times$384$\times$192	768$\times$512$\times$203	0.106	122400	28800	*P*	*P*
Verification	576$\times$384$\times$192	768$\times$512$\times$203	0.106	3600	25200	*I*	*P*

The driver simulation is designed to produce a rural boundary layer with a roughness length $Z_0=0.5$ m. Since this roughness length is of similar order of magnitude to the cell size in the LES, the use of a smooth wall with a wall function is not suitable. Therefore, the Macdonald et al. (1998) relation is used to estimate the parameters that reproduce $Z_0$ using an array of staggered cubes. With a packing density of 0.125 and block height of 3.2 m a roughness length of 0.5 m is obtained. The flow friction velocity is increased in the full-scale LES application to $U_{\tau \mathrm {LES}} = 0.1055$ m s$^{-1}$. The roughness length applied to the individual blocks is 0.05 m. The freestream velocity is $U_{\infty \mathrm {LES}}=1.94$ ms^−1^.

The velocity and Reynolds shear stress profiles show reasonably good agreement between the experiments and LES results (Fig. 4). The quasi-linear Reynolds shear stress profile of the LES data is expected by definition when the flow is in statistical equilibrium. The velocity profiles match well, except near the wall where the difference between flume and LES (smooth vs. rough-wall turbulent boundary layer) becomes evident.

The outflow plane from the driver simulation is used as input at the inlet of the verification simulation. The verification simulation uses inflow-outflow boundary conditions in the *X*-direction. Therefore, this resembles the water flume with the boundary layer flow upstream developing in the streamwise direction as it is transported over the model rig. The first 3,600 s are spin up, the data analysed are the next 25,200 s. To simulate the pollutant release upwind of the urban area, a continuous point source is introduced at the origin *X*=0 with strength $\dot{M}=1$ gs^−1^. The source is introduced directly in the advection-diffusion equation and does not affect the velocity field (no injection of fluid). To compare the flume and LES results, the concentrations are scaled by the source concentration $C_s$. Without $C_s$ in the LES, this is obtained by matching the mean concentration profiles at $X/\delta$=0.5. This scaling is permitted as the advection-diffusion equation is linear with concentration *C*.

## Results and discussions

### Scale-dependency of flume results

**Fig. 5:**
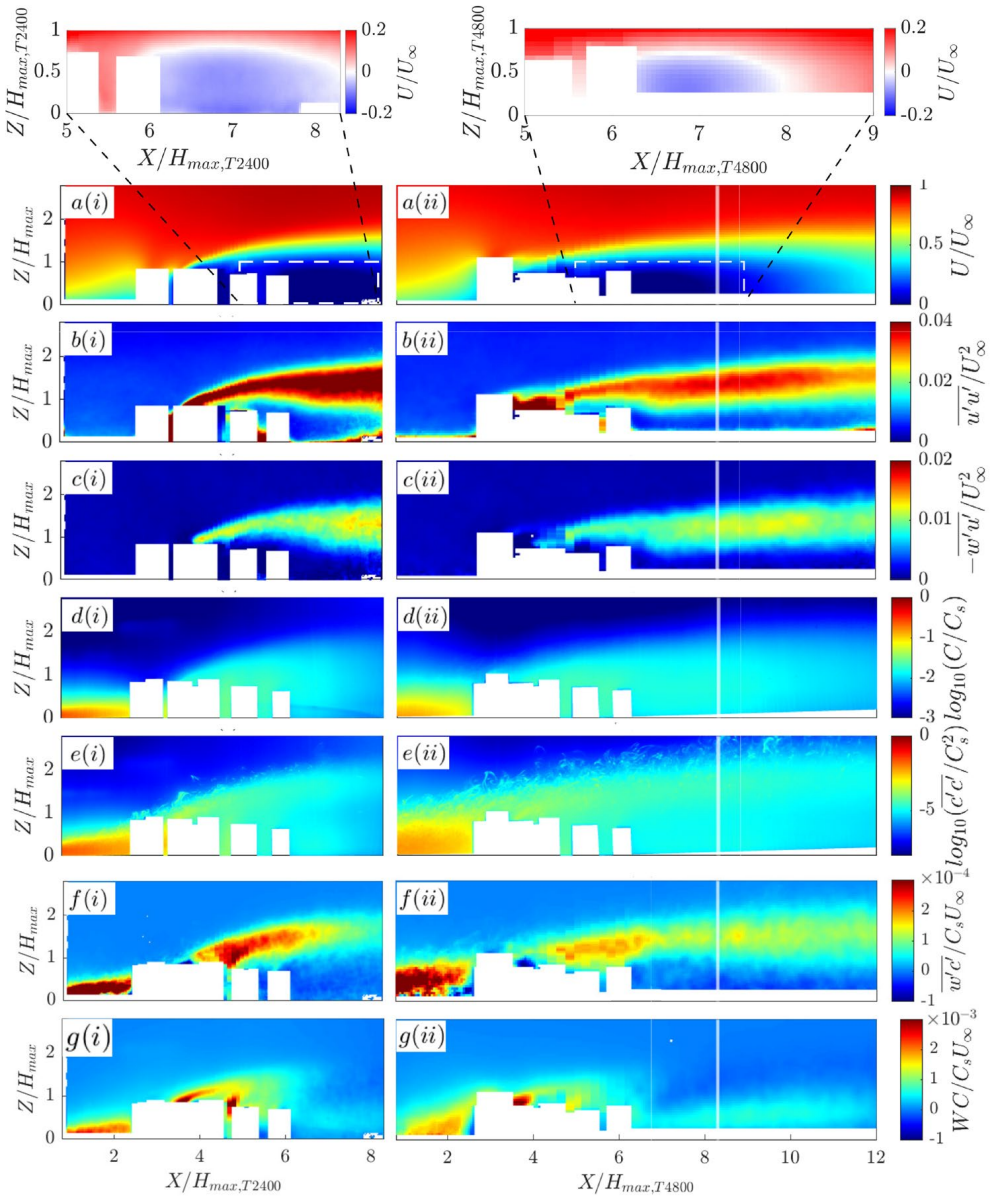
Water-flume contours for the (i) T2400 and (ii) T4800 cases (Fig. 2b,c) normalized by their respective length scales (Sect. 2.1), showing **a** mean velocity, **b** velocity variance, (c) Reynolds shear stress, **d** mean concentration, **e** concentration variance, **f** turbulent scalar flux and **g** advective scalar flux. Insets (**a**) show the recirculating flow in the cluster near-wake region. T4800’s vertical white lines indicate the outflow boundary for the T2400 case

The simplified tall-building cluster (without low-rise buildings) at two scales (T2400, T4800) is used to gain insight into the influence of geometrical scale on the velocity and scalar fields measured. The mean velocity, velocity variance, Reynolds shear stress, mean concentration, concentration variance, turbulent scalar flux and advective scalar flux maps are shown in Fig. 5 for both scales of model. To account for the scale difference, the streamwise and vertical axes are non-dimensionalized by the height of the tallest buildings in each model.

**Fig. 6:**
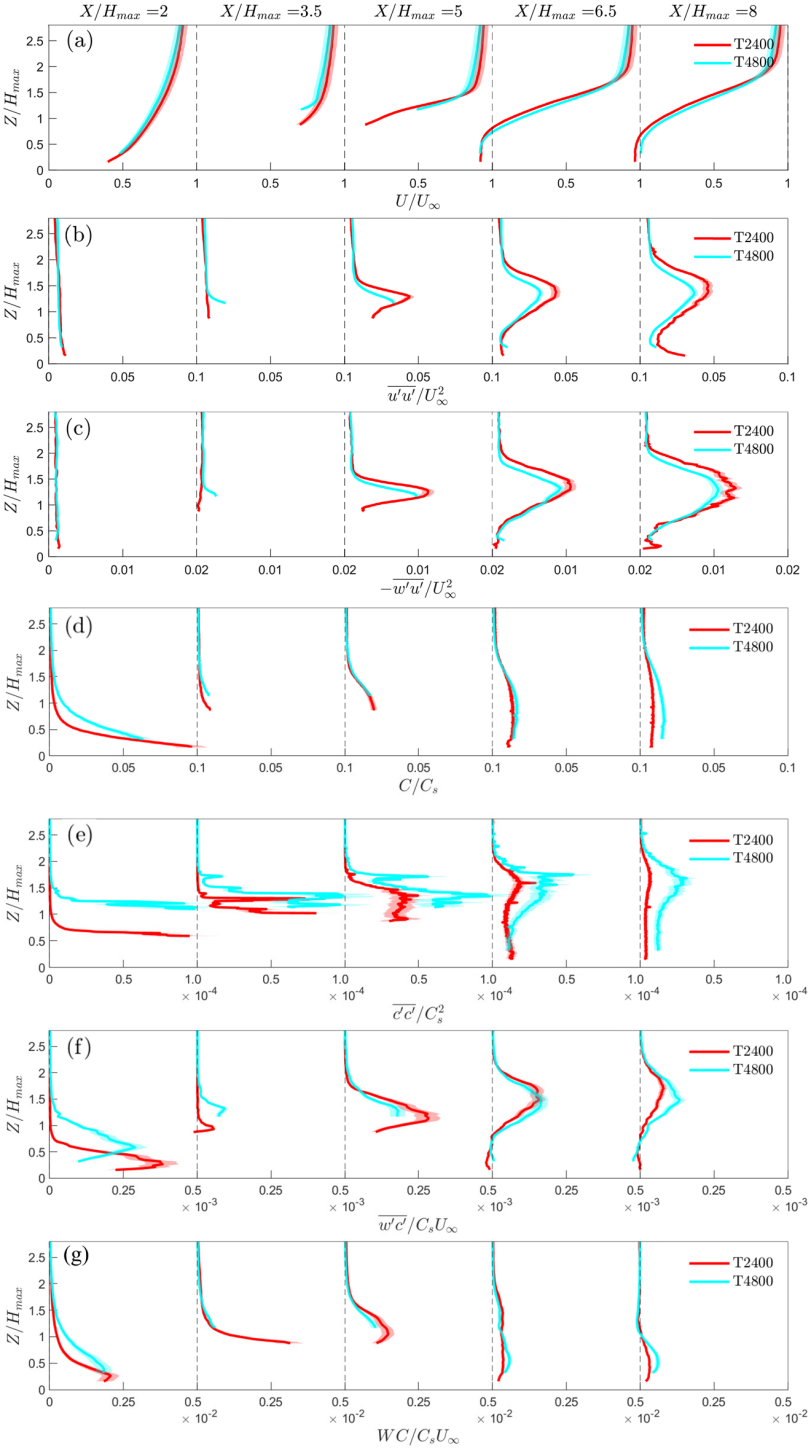
Water-flume vertical profiles for T2400 and T4800 normalized by their respective length scales (Sect. 2.1), showing **a** mean velocity, **b** velocity variance, **c** Reynolds shear stress, **d** mean concentration, **e** concentration variance, **f** turbulent scalar flux and **g** advective scalar flux extracted at regular streamwise intervals. Uncertainties (shading) are based on the sum of random and bias errors at 95% confidence interval (Appendices B, C)

In the mean velocity and velocity variance contours for the T2400 (Fig. 5a,b(i)), the growth and development of a rooftop shear layer originating from the tall building at approximately $X/H_{max,T2400}\simeq 4$ is evident, as is the recirculating flow region in the urban canopy layer and on the leeward side of the cluster ($X/H_{max,T2400}>$6). The same observations can be made for T4800 (Fig. 5a,b(ii)) but the recirculating flow region does not extend beyond $X/H_{max,T4800}>$8. The Reynolds shear stress contours (Fig. 5c) and vertical profiles (Fig. 6c) have good agreement with the peaks in the vertical profile corresponding to the rooftop shear layer, but T2400 has greater peak magnitudes than T4800.

The mean concentration and concentration variance results have similar characteristics for both T2400 and T4800, but the vertical profiles (Fig. 6d,e) show qualitative and quantitative differences with higher magnitudes at all streamwise location for T4800. Near the source, where the dye concentration is still very high, the dye response is no longer linear and secondary fluorescence creates a ‘halo’ effect that is noticeable in the flume measurements (Fig. 5d,e(i,ii)). Based on Vanderwel and Tavoularis (2014) and Baj et al. (2016), this local concentration bias is estimated to be up to 60%. However, this is expected to be negligible further from the source hence no correction is applied. The larger variability in the concentration contours also reflects the inherent uncertainty of the scalar transport that is dominated by a few large events (Fig. 5e). The corresponding profiles (Fig. 6e) are somewhat ‘fuzzy’, as is typical in highly intermittent scalar fields measured for limited duration with thin dye plumes. It is attributed to non-Gaussian, highly skewed probability density functions of the scalar fluctuations (Vanderwel and Tavoularis 2016).

The turbulent scalar flux (Figs. 5f, 6f) appears to be concentrated along the rooftop shear layer, suggesting the presence of instabilities that contribute significantly towards the vertical exchange of scalars. Near the building cluster, the advective scalar flux magnitudes (Figs. 5g, 6g) are higher than the corresponding turbulent scalar flux components (Figs. 5f, 6f) due to strong updrafts and downdrafts. Downwind of the building cluster, the magnitudes of the advective scalar flux decreases quickly and do not appear to be associated with the rooftop shear layer which persists for much further downstream distances. The contribution of the turbulent scalar flux to the total scalar transport increases in this shear-dominated region.

#### Implications of model scale for flume experiments

The size of the flume facility and domain size of interest influences the choice of geometrical scales for hardware models. However, the scale choice has several consequences on the velocity and scalar fields measured that need to be considered when interpreting the results.

In this experimental campaign, the dye source size and position relative to the model centre are fixed. Changing the model scale changes the effective size and location of the dye plume source relative to the building height. This leads to a larger incoming plume width relative to the building height for T4800 *cf.* T2400, which is evident in the concentration statistics (Fig. 5d,e) and the corresponding vertical profiles at $X/H_{max}$=2 (Fig. 6d,e), where the T4800 profiles have a vertical offset *cf.* T2400. This likely contributes to the discrepancies in the scalar statistics, where the means and variances are larger for T4800 *cf.* T2400.

Based on the concentration statistics (Fig. 6), the concentration variance is the most sensitive variable to the effects of scale. There are implications for air quality modelling where peak-to-mean concentration levels are of interest. When we non-dimensionalize the concentration variance using the building height and source properties following Sessa et al. (2018), the vertical profiles of the cluster near-wake flow do not collapse. While meandering is the main source of concentration fluctuations in Fackrell and Robins (1982b) much simpler application involving an elevated source in smooth-wall turbulent boundary layers, the complex/realistic urban landscape in this study introduce nonlinear effects on the concentration fluctuations. The source size, relative to the turbulence lateral integral scale, has a large effect on the concentration fluctuations (Fackrell and Robins 1982a), but it is unclear what effect the interaction of a ground source with a complex urban landscape has on the concentration fluctuations. We are unaware of any empirical expression in the literature that will normalize the concentration statistics in complex urban flows. Accounting for the model scale for concentration fluctuations requires consideration of the lateral turbulence scales near the ground and investigating the nonlinear interaction of these scales with the urban landscape. These are non-trivial problems that are currently being investigated.

The change in model scale makes the laser sheet proportionally thicker (in the lateral *Y* direction) for T4800 than T2400, hence covering a wider non-dimensional *Y*-section within the urban model, thus expanding the spatial averaging effect within the derived statistics. In very heterogeneous urban settings (as this study), flow and scalar fields have a large spatial variability so the laser sheet thickness impacts the processes sampled. For example, the tall building at $X/H_{max,T2400}\simeq 3$ does not affect the measured flow field for T2400 because it is in front of the laser sheet (Fig. 5a,b(i)), while the same building at $X/H_{max,T4800}\simeq 3$ has an effect on flow within the laser sheet for T4800 resulting in strong effects on the flow statistics in the measurement plane (Fig. 5a,b(ii)). This highlights that in geometrically complex settings and at small model scales, a relatively thick laser sheet can have a significant impact on what the measurements represent. There is a practical limit to the minimal width of the laser sheet (typically around 1 mm) after taking into account in- and out-of-plane movements of the seeding particles. Hence, using a very small scale model that is expected to generate highly three-dimensional flow features can lead to significant spatial averaging effects which needs to be considered when interpreting the results.

#### Influence of the incoming boundary layer conditions

As both the mean velocity and turbulence properties of the incoming flow impinging on the T2400 and T4800 models effectively differ, comparing the flow fields of these two cases is expected to reveal the effect of immersing the tall buildings in different parts of the approach boundary layer flow. With a change in fetch from rural-to-urban, the first buildings will typically extend into the logarithmic region of the oncoming rural atmospheric boundary layer. In the flume, the depth of the logarithmic region of the incoming flow extends to $\sim$18 mm (Appendix B), which translates to $\sim$43 m (T2400, LT2400) and $\sim$86 m (T4800), respectively, in full scale. This has the following implications for the cases investigated:

- **T2400**: All tall buildings extend beyond the logarithmic region of the inflow boundary layer.
- **T4800**: All tall buildings are within the logarithmic region.

The mean velocity, velocity variance and Reynolds stress (Fig. 6a–c) match well at all streamwise locations with only minor differences in the magnitudes which show that the velocity shear created at the rooftop of the tall building dominates over differences in the background turbulence in the outer and logarithmic layer. This hypothesis is further supported by the well-matched turbulent scalar flux profiles (Fig. 6f). Near the source (at $X/H_{max}=$2), there is a mismatch in the height of the peak due to the difference in the relative source size (Sect. 3.1.1). These profiles align after the oncoming flow encounters the first tall building in the measurement plane ($X/H_{max} \ge 5$), which illustrates the dominating influence of the tall building.

Insights into the slightly larger magnitudes of velocity variance and Reynolds stress for T2400 *cf.* T4800 can be gained by comparing the results of our study to cuboid flows literature. Wang et al. (2006) show that as the boundary layer thickness increases, the base vortices are enhanced, leading to stronger upwash flow that weakens the downwash effects of the free-end shear layers. This leads to a decrease in the Reynolds stresses near the ‘ground’ and an increase near the free-end of the cuboid (i.e., ‘roof’). Given the T4800 buildings are shorter (*cf.* T2400, w.r.t. $\delta _\mathrm {EXP}$), the incoming boundary layer is proportionately deeper. This could explain the lower Reynolds stresses observed throughout the entire building height in the near-wake flow region of T4800 ($Z/H_{max}<$1 in Fig. 6b,c). The lack of high Reynolds stresses near the rooftop may be attributed to several differences between Wang et al. (2006) and our study: (1) the buildings in the Beijing models are geometrically much more complex and form a cluster, (2) the cuboid height-to-diameter aspect ratio ($AR=5$) in Wang et al. (2006) is slightly larger than in this case ($AR<3$), and (3) the boundary layer thickness is less than the cuboid’s height in Wang et al. (2006).

The vortex lines of individual buildings are expected to be similar to those observed by Tanaka and Murata (1999) with a wide lateral spread of the legs that extend several diameters from a round cylinder of comparable AR. Coupled with the arrangement of the buildings in close proximity to each other, this is expected to result in the vortex lines of different buildings interacting with each other thus creating a much more complex flow scenario than a single cuboid (Wang et al. 2006). Nicolai et al. (2020) demonstrate that flow around a cluster of round cylinders has similar features to a single cylinder, with free-end shear layer created along the roof-line and mixing layers formed at the sides of the cluster. The cluster porosity breaks up the coherence and the wake flow is subject to greater turbulence. As city flow combines the effects of individual buildings, the change in the oncoming boundary layer thickness is expected to influence the interactions of all these vortex lines, resulting in the observed differences in the near-wake flow between T2400 and T4800.

### Flume experiment and LES

**Fig. 7:**
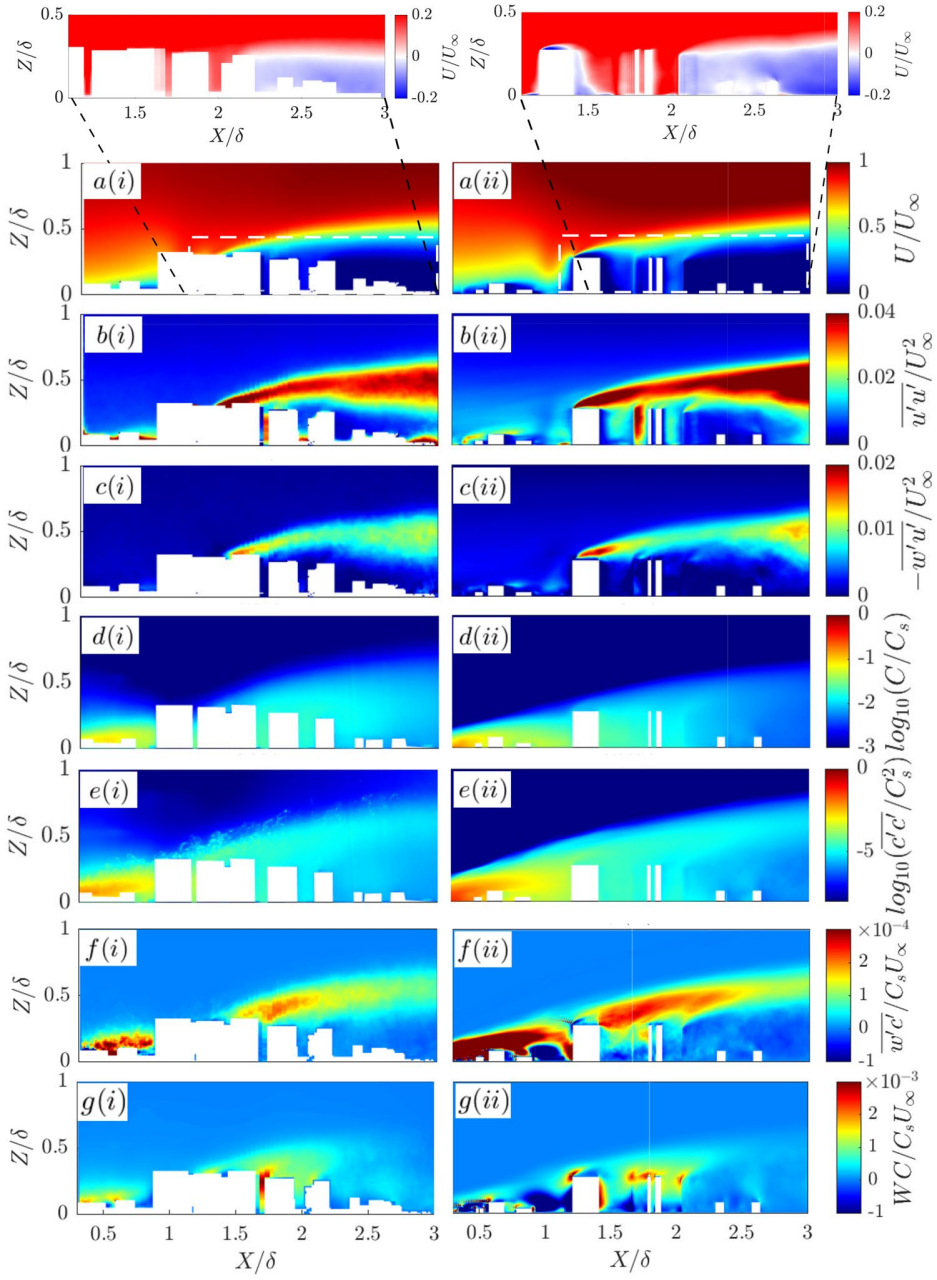
Contours for the LT2400 case (Fig. 2a,d) showing **a** mean velocity, **b** velocity variance, **c** Reynolds shear stress, **d** mean concentration, **e** concentration variance, **f** turbulent scalar flux and **g** advective scalar flux for (i) flume and (ii) LES data normalized by their respective length and velocity scales (Sects. 2.2, 2.3). Insets (**a**) show the recirculating flow on the roof of the tall building and in the cluster near-wake region

**Fig. 8:**
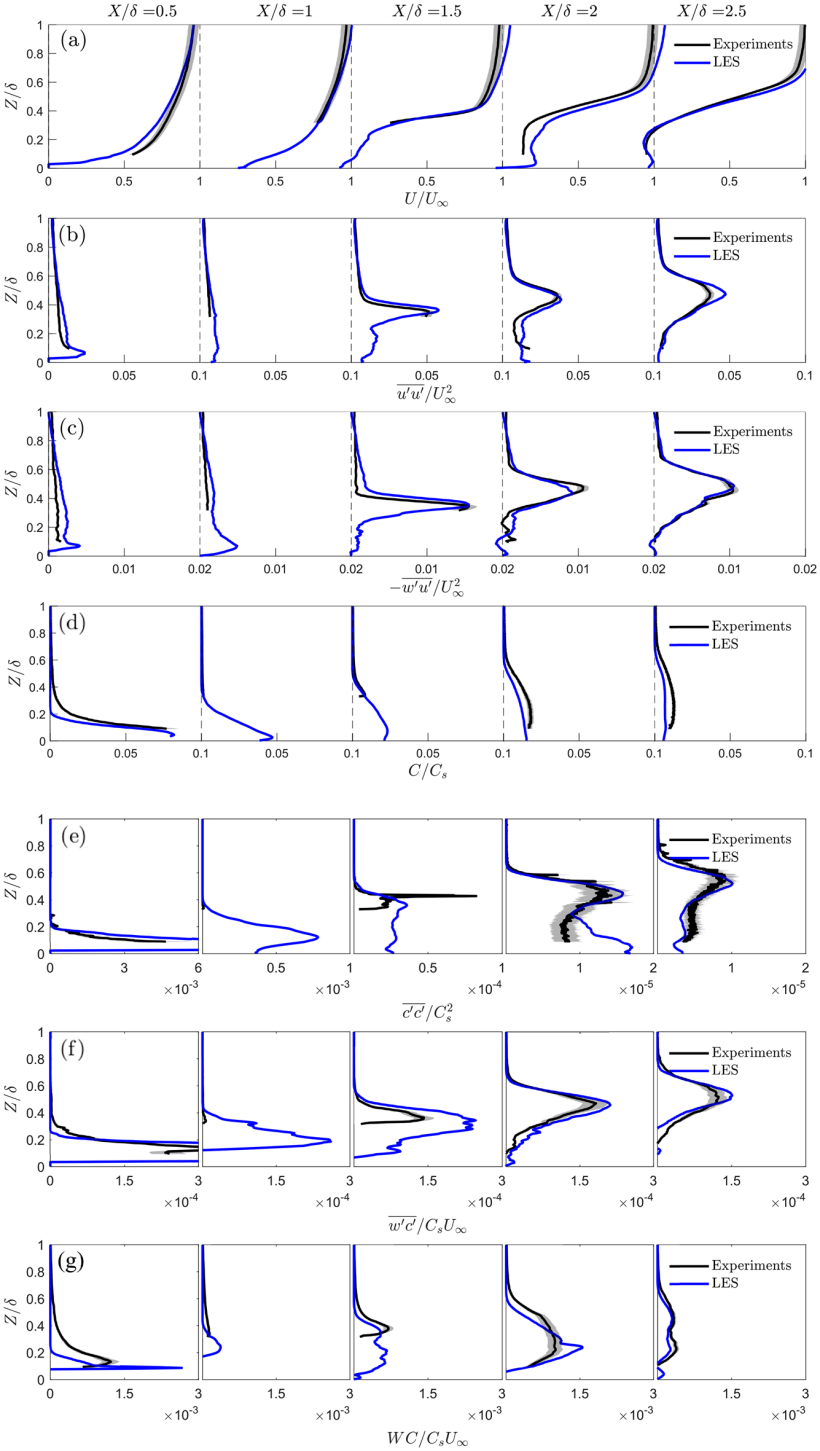
Water-flume and LES vertical profiles for the LT2400 case (Fig. 2a,d) normalized by their respective length and velocity scales (Sects. 2.2, 2.3), showing **a** mean velocity, **b** velocity variance, **c** Reynolds shear stress, **d** mean concentration, **e** concentration variance, **f** turbulent scalar flux and **g** advective scalar flux extracted at regular streamwise intervals. Uncertainties (shading) are based on the sum of random and bias errors at 95% confidence interval

Flume and LES data for the LT2400 (Fig. 2a,d) case are analysed to cross-verify the different methodologies. Comparisons of flow and dispersion variables are presented as both maps (Fig. 7) and vertical profiles (Fig. 8) with the dye source location defined as the origin in both. Note, LES data are available for the complete plane, but the flume data have a reduced field-of-view when buildings in front of the laser sheet block the view into some street canyons.

The flow fields for the flume and LES are remarkably similar even though the flume experiments and LES are performed at different scales with slightly different incoming flow conditions (Fig. 4). The mean velocity contours (Fig. 7a) both indicate the development of a strong velocity shear zone from the rooftop of the tall building (rooftop shear layer) farthest upstream ($X/\delta \simeq 1.25$). The velocity variance and Reynolds shear stress contours (Fig.  7b,c) also show two clear boundaries originating from the roof of the first tall building, consistent with the location of the strong velocity shear generated from the rooftop. The flume and LES agree well with each other in both location and magnitude. The separated shear layers from the roof defines the boundaries of the rooftop shear layer (Sect. 3.2.1).

At $X/\delta$=1, a tall building located in front of the laser sheet is masked out in the flume results (Fig. 7a(i)). For the corresponding LES results (Fig. 7a(ii)) masking is unneeded, allowing locally accelerated flow redirected to the side of the building to be observed. At $X/\delta$=2, the region near the ground has slightly larger differences for the vertical profiles of the velocity statistics (Fig. 8a–c). This is attributed to larger localised experimental uncertainty as the region is surrounded by two tall buildings and near-ground measurements are notoriously difficult to acquire with high confidence with the PIV technique. Challenges include shadows, laser reflections and buildings in the foreground blocking the camera’s line-of-sight, which can lead to missing velocity information in this region. In the near-wake of the tall-building cluster at $X/\delta \approx$ 2.5, backward flow is observed to extend from the ground to roof level of the tall building (72 m; Fig. 8a and Fig. 7a insets). Reattachment is not observed in the flume or LES domain.

The main features of the observed concentration distribution within the rooftop shear layer are qualitatively quite well replicated in the LES, whereas the predicted concentration magnitudes do not agree as well as the velocity statistics. This is attributed to the challenges of measuring mean concentrations close to the source in the flume, a thicker averaging plane because of the laser sheet thickness and the difficulty in appropriately scaling the LES concentration statistics. For both the flume and LES, the vertical extent of the mean concentration plume grows strongly after encountering the first tall building. There is a slight difference in the vertical half-width, more noticeable at $X/\delta$=3 (Fig. 7d). The corresponding vertical profiles (Fig. 8d) show the flume measurements always have higher mean concentrations than the LES.

Spatial patterns and magnitudes of the concentration variance (Fig. 7e) agree well in most locations that are unaffected by masking, except for the small region within the urban canopy layer at $X/\delta$=2 (Fig. 8e). This region matches poorly because of its proximity to the tall buildings. We note that the concentration variance has a strong dependence on the source properties (Fackrell and Robins 1982b), and the difference in scale between the flume and LES means this can introduce additional uncertainties.

The turbulent scalar fluxes (Fig. 8f) show good agreement except for $X/\delta<$0.5, where the LES predicts much higher peaks, and at $X/\delta =$1, where negative fluxes can be observed due to redirected flow by the tall building outside the plane (e.g. Fig. 7a(ii)). The vertical location of the peak in the turbulent scalar flux originating from the tall building is consistent with the peaks in velocity shear, velocity variance, Reynolds stresses, mean concentration and concentration variance, suggesting the first tall building encountered by the incoming flow has the largest overall effect on the incoming flow. The advective scalar flux (Figs. 7g, 8g) is much higher within the canopy layer due to building-induced vertical motions, and this is particularly evident in the LES results (Figs. 7g(ii), 8g) which is not constrained by optical access. From the LES, it is also clear that the advective scalar flux dominates the total scalar transport within the UCL where large magnitudes of vertical and lateral velocities are expected.

Given the differences in scale and incoming flow profiles of the flume and LES methods (Sect. 2; Fig. 4), one may have expected differences in the flow structures. However, only relatively small differences are observed indicating the flow dynamics induced by the roughness elements of the urban model overwhelms any differences between the inflow conditions and that the flow is Reynolds number independent.

#### Rooftop shear layer

The rooftop shear layer is responsible for enhanced momentum and scalar transport in all the model geometries. In studies of regular arrays of roughness elements (e.g. Tomas et al. 2017), ‘rooftop shear layer’ is referred to as an ‘internal boundary layer’, but our study has sparse and non-uniform buildings, hence we do not use that terminology.

For the detailed LT2400 case (Figs. 7, 8), when the incoming flow impacts the first tall building within the laser sheet plane at $X/\delta =$1.25, the flow diverges around the side and top of the building. The LES results clearly show an initial separation at the leading edge, reattachment near the trailing edge of the rooftop, and backward flow on the leeward side of the building (Fig. 7a(ii)). These features are obscured in the flume results (Fig. 7a(i)). The literature suggest no reattachment on the sides and roof of isolated buildings with height larger than its streamwise length (Oke et al. 2017). However, the urban model in the current study is much more complex, with upstream neighbourhood buildings (e.g. at $X/\delta$=1) introducing advective fluxes, flow vortices and turbulence to the flow which can encourage reattachment. The velocity profiles (Fig. 8a) have strong shear and inflection points starting from $X/\delta \ge$1.5. These suggest the presence of Kelvin-Helmholtz instabilities, which is supported by peaks in the vertical profiles of the velocity variance and Reynolds shear stress (Fig. 8b,c) that grow in width with downstream distance. The increased turbulence in the rooftop shear layer is expected to improve vertical scalar bulk transport and contribute significantly to the turbulent fluxes in the roughness sublayer. This is supported by higher turbulent scalar fluxes occurring in the same region for all cases (Figs. 7f, 5f).

The magnitudes of the Reynolds shear stress in the rooftop shear layer agree remarkably well with Hertwig et al. (2019) wind-tunnel results from low and (isolated) tall buildings, despite differences in the urban geometry, model scale and experimental method. One notable difference is the vertical location of the peak, whilst Hertwig et al. (2019) finds the rooftop shear layer develops just below the tall building height here the rooftop shear layer develops at and above the building height. This may be because of the differences in both urban morphology (i.e., isolated vs. cluster of tall-buildings), experiment inflow and boundary conditions (e.g. $H_{max}/\delta \sim 1$ in Hertwig et al. (2019)), and sparser measurement points from the Laser Doppler Anemometry in Hertwig et al. (2019) compared to PIV. The similarities in Reynolds shear stress magnitude suggest the development of the rooftop shear layer due to the tall building is not very sensitive to the incoming flow conditions. The absence of a secondary peak in the Reynolds shear stress near the ground (as observed by Hertwig et al. (2019)) can be attributed to the porosity of the cluster which reduces the strength of the recirculating flow in its wake.

#### Development of the concentration plume

**Fig. 9:**
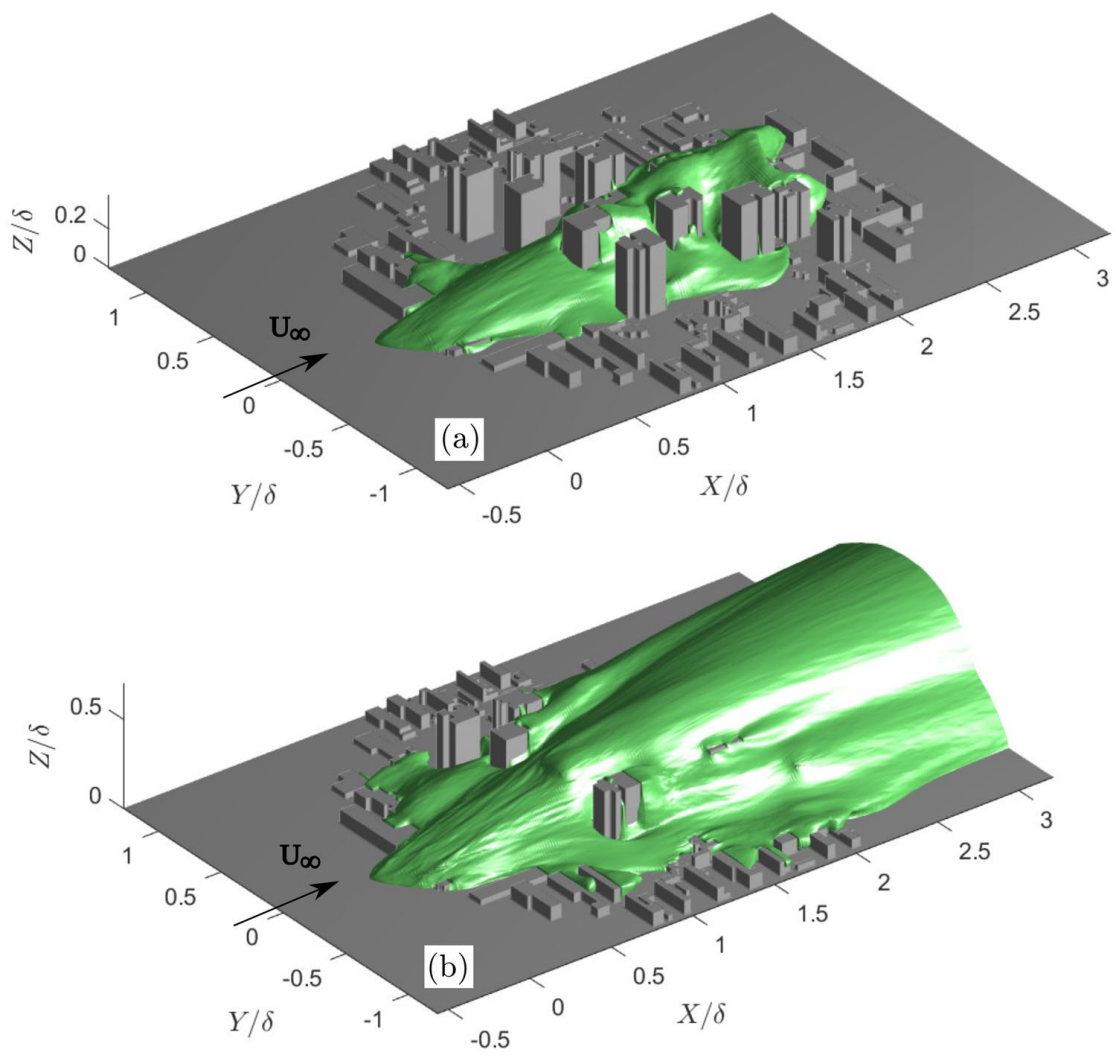
Isosurfaces of the concentration plume (green) extracted from the LES using isovalues of **a** ${C/C_s}=10^{-2}$ and **b** ${C/C_s}=10^{-3}$

The 3D development of the concentration plume can be examined by extracting the isosurfaces of concentration from the LES results for selected isovalue thresholds (Fig. 9). The low-rise canopy upwind of the tall buildings introduces a strong lateral spread of the plume along the street canyons oriented in the *Y* direction (Fig. 9). Pollutants can re-circulate in these lateral-canyons and are detrained vertically out of the lower canopy layer before impacting the tall-building cluster further downwind (Fig. 9b). As the plume hits the tall building cluster, there is a rapid increase in the vertical spread of the plume, and the wakes of the tall buildings located furthest upwind interact with downstream low- and high-rise buildings to further alter the cluster near-wake dynamics and plume geometry. The vertical and lateral spread of the plume in the RSL can be attributed to strong updrafts, downdrafts and side-drafts around the tall buildings (expanding the depth of the UCL), as well as flow channelling within the street canyons in the urban canopy. The changes in the mean flow direction and magnitude within the RSL contribute to the advective scalar flux (Fig. 7g). The total scalar transport is also affected by turbulence generated by the low- and high-rise buildings which contributes to the turbulent scalar flux component (Fig. 7f). For accurate prediction of peak concentration levels, it is necessary to consider both advective and turbulent scalar flux components.

### Effects of low-rise neighbouring buildings

**Fig. 10:**
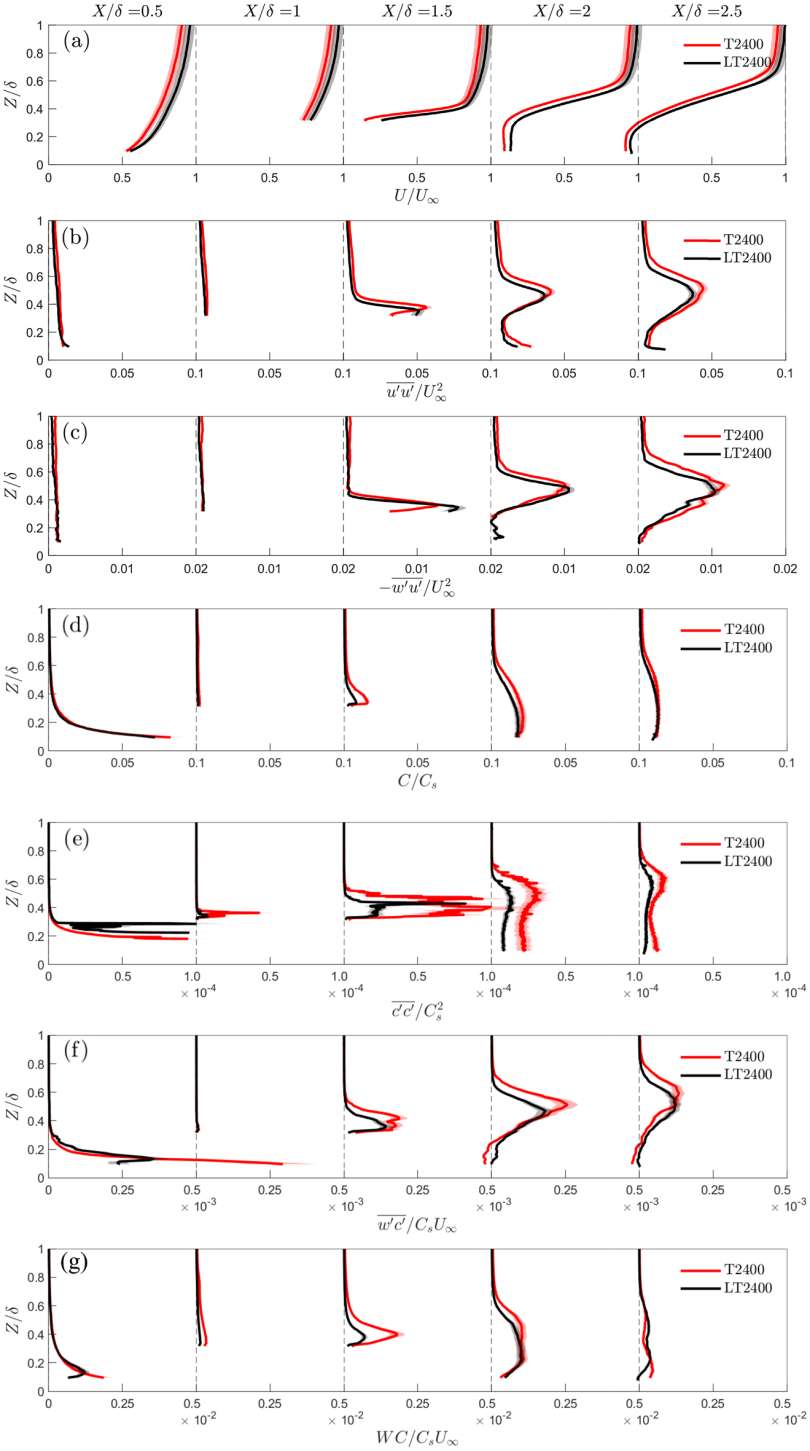
Water-flume vertical profiles for the T2400 and LT2400 cases, showing normalized **a** mean velocity, **b** velocity variance, **c** Reynolds shear stress, **d** mean concentration, **e** concentration variance, **f** turbulent scalar flux and **g** advective scalar flux extracted at regular streamwise intervals. Uncertainties (shading) are based on the sum of random and bias errors at 95% confidence interval (Appendices B, C)

The influence of the low buildings on the flow is analysed by comparing the simplified tall-buildings-only (T2400) and more realistic with low-rise building (LT2400) cases. The mean velocity profiles generally agree well with each other, with the LT2400 case having slightly higher streamwise velocities at all locations compared to the T2400 case (Fig. 10a). This is attributed to the presence of the low-level canopy in LT2400 which displaces fluid vertically and to the sides due to blockage effects. The velocity variance and Reynolds shear stress show similar trends, but the peak rooftop shear layer from the tall buildings is just slightly higher and larger in magnitude for the T2400 case (Fig. 10b,c). The low-rise canopy in LT2400 has the effect of increasing the zero-plane displacement, hence the incoming flow experiences a relatively shorter tall-building for LT2400 *cf.* T2400, thus inducing a lower shear layer.

The mean concentration profile trends (Fig. 10d) agree well in most streamwise locations except near $X/\delta =$1.5 and 2 where higher mean concentrations are seen in the T2400 case *cf.* LT2400 (Fig. 10d). This is attributed to higher level of vertical advection of dye in T2400 (Fig. 10g), which is consistent with the higher shear layer and greater magnitudes of velocity variance and Reynolds shear stress *cf.* LT2400 (Fig. 10c). The presence of low-level canopy in LT2400 also promotes the advection of dye in the lateral direction at the street-level (channelling and branching of the plume along streets and through intersections) which can locally reduce the mean concentration levels. While this is not observed in Fig. 10g due to a lack of optical access, the isosurfaces of the concentration plume from the LES results clearly shows significant advection of dye laterally along the low-rise street canyons (Fig. 9). The concentration variances have large quantitative differences between the two cases. T2400 has similar trends but systematically much larger magnitudes at $X/\delta \ge 1$ *cf.* LT2400. The turbulent scalar fluxes have similar characteristics to the Reynolds stresses, with the T2400 peaks elevated at all distances from the source. The T2400 peak magnitude is also higher due to larger concentration fluctuations (larger concentration variances).

The observation of significant flow advection in the lateral direction (Fig. 9) shows the oncoming flow (and concentration plume) can be significantly altered by the low-rise buildings before it encounters the tall building. Even though the results show tall buildings dominate to a large degree the flow structure in the wake, the effect of the low-rise buildings on the velocity and concentration statistics is non-negligible considering they occupy only a comparatively small portion of the patch (Fig. 2a). Differences in the T2400 and LT2400 velocity and concentration statistics are quantitatively comparable to the differences in the T2400 and T4800 cases (Fig. 6). This suggests the extent of the influence of the low-rise buildings is similar to the impact of the choice of scale on the overall flow field, and clearly not as important as the momentum and scalar fluxes associated with the rooftop shear layer.

## Conclusions

Pollutant dispersion around a realistic tall-buildings-only cluster (based on a Beijing neighbourhood) is investigated at two scales (T2400 and T4800) using PIV- and PLIF-based flume measurements. This is complemented with an additional case consisting of low-rise buildings surrounded by the tall-building cluster (LT2400), investigated using flume and LES analyses.

Flume observations at two scales (T2400, T4800) find the rooftop shear layer dominates the vertical momentum and scalar fluxes. The magnitude and vertical location of the corresponding peak agree well, with minor differences attributed to differences in the incoming boundary layer thickness (relative to the building height) due to the choice of model scale. Concentration levels decrease with downstream distance due to advective and turbulent scalar transport and at $X/H_{max}=8$ it has reduced to 1.7% of the source concentration for T4800 and 0.9% for T2400. The concentration variance is found to be highly sensitive to the scale choice due to changes in the width of the incoming dye plume. Scale also influences the laser sheet thickness (i.e., relative to building scale), which impacts the processes that are sampled in a very heterogeneous urban settings (as this study). This increases the spatial variability due to significant spatial averaging effects. The influence of scale on the cluster near-wake flow highlight critical details for future flume studies:

1. Care should be taken to scale the source size and location with respect to the height of the building inducing the rooftop shear layer.
2. Laser sheet thickness should ideally be an order of magnitude less than the characteristic length scale of the model to reduce effects of the spatial-averaging of three-dimensional flow fields.
3. The scale of the building heights relative to the depth of the log-law region of the incoming boundary layer and the inclusion of low buildings in the urban model are important to capture the peaks in momentum and scalar fluxes accurately.

The velocity and concentration statistics for the LT2400 case have good agreement between flume and LES results in most parts of the analysis domain. Notably, the development of the rooftop shear layer and its associated vertical momentum and scalar fluxes have remarkable agreement in both magnitude and position of the peak despite differences in the scale and incoming flow conditions. The concentration levels decrease to approximately 1.1% of the source concentration in the experiment and 0.5% for LES at $X/H_{max}=8$. Minor differences in the concentration statistics are attributed to the variability introduced by differences in the scale and inflow profile. The magnitude of the vertical momentum flux also agrees well with the Hertwig et al. (2019) results for a low and (isolated) tall building. Only the vertical location of the peak differed slightly between the two studies despite differences in urban morphology, model scale and experimental method. It is concluded that the inflow conditions do not appear to be crucial in the development of the rooftop shear layer.

Flume observations of LT2400 and T2400 reveal non-negligible effects of the low-rise buildings on the velocity and concentration statistics, with LT2400 having higher streamwise velocities above the UCL due to fluid displacement by the low-level canopy, and lower concentration levels in the measurement plane due to increased advection of the plume in the lateral direction at the street-level. These differences are quantitatively comparable to those observed between T2400 and T4800, and it is concluded that the extent of the influence of the low-rise buildings is similar to the impact of the choice of scale on the overall flow field.

There are a number of key findings with general applicability to studies of dispersion in real urban areas. Firstly, tall buildings expand the depth of the UCL and introduce strong flow disturbances in the RSL, which dominate over differences in initial flow conditions and influences of the low-rise buildings. We conclude that for urban canopy model parametrisations (as needed for weather and climate modelling), inclusion of the impact of building-height heterogeneity is clearly important. Secondly, below the mean canopy height, advective fluxes in the lateral direction are significant, which can be difficult to model as they are geometry dependent. Thirdly, in regions with strong flow shear, the turbulent scalar transport is larger than the advective scalar transport. These findings show complex (but realistic) urban areas introduce additional challenges that need to be addressed in model prediction. This will be especially important if models that lack building-induced flow effects parametrisations are used to simulate dispersion.

Further studies on the impact of building-height heterogeneity that allow for systematic testing are clearly required to develop and/or enhance model parametrisations of roughness sublayer or urban canopy layer flow fields. Specific test cases based on realistic geometries, as presented in this study, are essential to evaluate such parametrisations. The data derived here, and the clear need for future studies of this type, are critical to making weather and climate model predictions more realistic over urban areas, with implications for other aspects of urban modelling (e.g. dispersion or air quality simulations). Further studies in the Beijing tall-building cluster will address the impact of wind direction and the implications for the interaction between the wakes of tall buildings of different heights.

## Data Availability

Data published in this article are available from the University of Southampton repository at 10.5258/SOTON/D2217.

## A Determination of the friction velocity in the flume

The friction velocity in the water flume, $U_{\tau \mathrm {EXP}}$, is determined using two methods. First, data are fit to the log-law equation for smooth walls: $U^+=(1/\kappa ) ln(Z^+)+B$ where $U^+=U/U_{\tau \mathrm {EXP}}$ and $Z^+=ZU_{\tau \mathrm {EXP}} /\nu$. Using $\kappa =0.4$ for the von Kármán constant and B=5 for the smooth-wall intercept. The calculated friction velocity is $U_{\tau }$=0.019 m s$^{-1}$.

Second, the total stress is calculated at the plateau of the Reynolds shear stress profile in the overlap region of the boundary layer (Fig. 4) using: $U_{\tau \mathrm {EXP}}=\sqrt{-\overline{u\prime w\prime }}$ under the constant stress assumption. The friction velocity from this method is $U_{\tau }$=0.0156 m s$^{-1}$.

The average of both is taken as the friction velocity, i.e., $U_{\tau \mathrm {EXP}}$ = 0.0173 m s$^{-1} \pm 10\%$. The depth of the log-law region is estimated to be within the range 3 mm $\le$ Z $\le$ 18 mm ($0.04< Z/\delta _\mathrm {EXP} < 0.22$).

## B PIV processing and uncertainty analysis

All image processing procedures for PIV use LaVision’s Davis 8.4.0 software (LaVision GmbH 2019). To pre-process the particle images, the ‘subtract time filter’ is set to 5 images and ‘subtract sliding background scale’ set to 8 pixels. Geometrical masking is performed on the measurement plane to remove buildings that block the camera’s view and in regions with unavoidable laser reflections. Multi-grid two-pass cross-correlation analysis is performed on the PIV images using a 50% overlap ratio. The final interrogation window size is 24 $\times$ 24 pixels. This translates to a vector resolution of $R_{PIV}$=0.8 mm.

For vector post-processing, Davis 8.4.0’s ‘allowable vector range’ is used to remove spurious vectors if above 12 pixel shifts. Vectors with correlation value below 0.7 are deleted, and the ‘universal outlier detection’ routine is used to remove vectors if residual is above 2 in a 5$\times$5 filter region. The neighbourhood interpolation scheme is used to fill the missing vectors. Spatial smoothing is performed to generate the final vector fields.

The main source of systematic error leading to velocity bias is expected to arise from the image scaling procedure. It is estimated to be $\sim$1.3%. For each case, 2,000 vector fields are used to obtain the velocity statistics. The standard deviation (turbulence intensity) of the streamwise fluctuating velocity in the freestream is 1.2%. For the 1:2400 (or 1:4800) scale models, the total measurement time at full-scale is 21.7 h (or 43.4 h) and the temporal spacing between consecutive vector fields is 39 s (or 78 s).

## C PLIF processing and uncertainty analysis

For PLIF, the fluorescence intensity measured at every pixel of the camera is expected to be a linear function of the local dye concentration multiplied by the local laser intensity. In this PLIF setup, the local laser intensity is constant and any pulse-to-pulse variations in the laser power are accounted for by monitoring the energy of each laser pulse with an energy monitor ($E_\mathrm {ref}$). The calibration procedure obtains the coefficients of the linear mapping function from fluorescence intensity to local concentration for each camera pixel.

A background measurement is obtained with $C_{0}$=0 mg L$^{-1}$. Two small calibration tanks with two known concentrations of the dye ($C_{1}$=0.03 mg L$^{-1}$ and $C_{2}$=0.05 mg L$^{-1}$) are sequentially inserted into the water flume and aligned with the laser sheet. Calibration images of the uniform concentrations ($I_0, I_1$ and $I_2$) are captured and post-processed to calculate the calibration coefficient, i.e., $A_{cal}=(A_{1}+A_{2})/2$, where $A_{i=1,2}=(C_{i=1,2}-C_{0})/(I^c_{i=1,2}/E_\mathrm {ref}-I_{0})$. Here, $I_i^c$ represents the image intensity in the calibration images corrected for light attenuation along the light path and secondary fluorescence (Vanderwel and Tavoularis 2014), and $E_\mathrm {ref}$ accounts for pulse-to-pulse variations in the laser power captured by the energy monitor of the laser. The final concentration field is obtained by applying the calibration coefficient to the raw PLIF image, *I*, via $C=(I/E_\mathrm {ref}-I_{0})A_{cal}$.

Using three known calibration concentrations (i.e., $C_{0}, C_{1}, C_{2}$) to determine the linear calibration coefficient permits an estimate of the uncertainty associated with this fit. The error associated with the mapping coefficients is estimated by defining the error term as: $\epsilon = (\epsilon _1+\epsilon _2)/2$, where $\epsilon _{i=1,2}=(I_{i=1,2}^c-I_{0})A_{cal}-C_{i=1,2}$, and computing the mean and standard deviation of the error term for the entire image excluding the borders. We estimate the potential bias error to be 0.8% and the potential random error as 5.3% (or 10.6% for a 95% confidence interval). The effects of secondary fluorescence may locally increase measurement uncertainties in some regions (Sects. 3.1 and 3.2) but are not explicitly corrected here as they require non-trivial methods to account for nonlinear effects. The spatial resolution of the PLIF results is $R_\mathrm {PLIF}$=0.0665 mm.

Geometrical masking is performed on the PLIF results. As the PLIF camera position relative to the buildings is different from the PIV setup, the buildings masked may appear to be slightly different because of the change in perspective. The masking procedure is performed on buildings in the laser sheet’s plane, at boundaries where the laser reflection cannot be avoided and on buildings (outside the laser sheet’s plane) that obstructed the camera’s line-of-sight.

The PIV vector fields and PLIF concentration fields have different coordinate systems and vastly different spatial resolutions due to the use of interrogation windows in PIV’s cross-correlation analysis. To calculate the joint velocity-concentration statistics, inhouse code maps the PIV and PLIF coordinates to a common coordinate system via linear interpolation. The mapped concentration field is sub-sampled to the PIV’s resolution after neighbourhood averaging using a box kernel with dimensions equivalent to $R_\mathrm {PIV}/R_\mathrm {PLIF}$, and selecting the concentration values at the points of the common coordinate system. The accuracy of the overlap of the coordinate system mapping is estimated to be within 3$R_\mathrm {PLIF}$. This is mitigated by the neighbourhood averaging and subsampling procedures and hence negligible. The combined uncertainty of the joint velocity-concentration statistics is estimated to lead to a systematic error of up to 11.9% in the measured scalar fluxes.
